# Severe Acquired Factor VIII Deficiency With Concomitant Lupus Anticoagulant: A Case Report

**DOI:** 10.7759/cureus.113229

**Published:** 2026-07-23

**Authors:** Tae Hoon Kim, Ammar Khawar, Jason Suh

**Affiliations:** 1 Department of Internal Medicine, Valley Health System, Paramus, USA; 2 Department of Hematology Oncology, Valley-Mount Sinai Comprehensive Cancer Care, Paramus, USA

**Keywords:** acquired hemophilia a (aha), autoimmune coagulopathy, factor viii inhibitor, lupus anti-coagulant, rare bleeding disorder

## Abstract

Acquired hemophilia A (AHA) is a rare autoimmune bleeding disorder caused by autoantibodies against factor VIII (F VIII). The coexistence of AHA with lupus anticoagulant (LAC) is incredibly uncommon and presents a significant diagnostic challenge, as both conditions prolong activated partial thromboplastin time (aPTT) and demonstrate incomplete correction on mixing studies. We report a case of a 79-year-old man with no prior bleeding history who presented with severe anemia and a retroperitoneal hematoma following a fall. Laboratory evaluation revealed markedly prolonged aPTT, severely reduced F VIII activity (<7%), and an exceptionally elevated inhibitor titer (1084 Bethesda units), consistent with AHA. Concurrently, anticoagulant testing was done with a positive LAC presence. Extensive workup failed to identify an underlying etiology. The patient was subsequently treated with rituximab and corticosteroids, resulting in a gradual decline in aPTT and inhibitor titers with clinical improvement. This case highlights the importance of comprehensive coagulation testing in patients with unexplained bleeding and prolonged aPTT, as the coexistence of prothrombotic and hemorrhagic conditions can obscure diagnosis and delay appropriate management.

## Introduction

Acquired factor VIII (F VIII) deficiency, alternatively known as acquired hemophilia A (AHA), is a rare and potentially life-threatening bleeding disorder caused by the development of autoantibodies that are directed against F VIII [1]. F VIII plays an important role in the coagulation cascade as it facilitates the activation of factor X, necessary for blood clotting. AHA typically arises later in adulthood, with an estimated incidence of one case per million annually [2]. A study showed that patients with AHA had a one-year survival rate of approximately 68% [3]. Diagnosis of AHA requires a high index of suspicion, especially in patients with isolated prolonged aPTT not corrected by mixing studies. The F VIII activity level must be low or absent, and the Bethesda assay would measure the strength of inhibition [3].

AHA can be caused by certain medications, including beta-lactam antibiotics, non-steroidal anti-inflammatory drugs, and heparin [4]. AHA may also occur in the postpartum setting or in association with autoimmune disorders or malignancies. However, in up to 79% of cases, no clear etiology is found. Clinically, patients commonly present with soft tissue hematomas, mucosal bleeding, or life-threatening hemorrhages in the absence of a bleeding history [2]. Antiphospholipid antibodies (aPL) bind to phospholipid-binding plasma proteins on cellular surfaces, triggering prothrombotic pathways that promote a hypercoagulable state. Lupus anticoagulant (LAC), a subtype of aPL, is paradoxically associated with thrombosis despite prolonging phospholipid-dependent coagulation assays, including the activated partial thromboplastin time (aPTT) [5]. Meanwhile, antiphospholipid antibodies (aPL) refer to a group of autoantibodies that affect phospholipid-dependent coagulation assays, leading to prolonged clotting times in vitro. These antibodies most commonly target phospholipid-binding plasma proteins such as β2-glycoprotein I and anticardiolipin antibodies [5]. This paradox arises because LAC enhances thrombin generation in vivo by concentrating prothrombin on phospholipid surfaces. The presence of aPL is a major risk factor for the development of antiphospholipid syndrome (APS), which is characterized by both arterial and venous thromboembolic events [6].

The co-existence of both AHA and LAC is exceedingly rare, with fewer than 20 documented cases. We report a case of a patient with no prior bleeding history who presented with a soft tissue hematoma, severe acquired F VIII deficiency, and positive lupus anticoagulant in the absence of a clear etiology.

## Case presentation

A 79-year-old Caucasian man with a past medical history of Parkinson’s disease and hypertension presented to the emergency department after suffering a fall, injuring his left leg. Vitals on initial presentation showed a heart rate of 112 beats per minute, blood pressure 130/88 mmHg, afebrile, respiratory rate 18 breaths per minute, and oxygen saturation 90% on room air. He denied any history of alcohol use, smoking, or illicit drug use. He did not have any significant family medical history and denied any past surgical history. His home medications included losartan 25 mg daily and carbidopa 25 mg-levodopa 100 mg daily. Table 1 highlights the patient's laboratory values at the time of admission. Computed tomography angiography (CTA) of the abdomen and pelvis was performed, which demonstrated a retroperitoneal hematoma of the left psoas and iliacus. He was transfused with one unit of packed red blood cells due to a low hemoglobin (Hgb) level of 6.6 g/dL, and the patient was admitted for further treatment and workup.

**Table 1:** Lab values at the time of admission

Parameter	Result	Reference Range
Hemoglobin (Hgb)	6.6 g/dL	Male: 13.5-17.5 g/dL
Hematocrit (HCT)	24.5%	Male: 40-54%
Prothrombin time (PT)	15.8 seconds	9.4-12.5 seconds
Activated partial thromboplastin time (aPTT)	103.9 seconds	25-37 seconds

Physical exam was most notable for a limited range of motion of the left leg with multiple blanching ecchymoses on all four extremities. Further hematologic workup results can be seen below in Table 2. An F VIII inhibitor profile was ordered, which showed an F VIII inhibitor titering assay (Bethesda) level of 1084 BU, and the patient was diagnosed with AHA. Further workup to identify the etiology of the acquired F VIII deficiency was initiated, including a CT chest scan, which found no occult masses. Antinuclear antibody and rheumatoid factor tests were performed to screen for autoimmunity, which were also unremarkable.

**Table 2:** Hematologic lab workup results

Parameter	Result	Reference Range
Factor XI activity level	<1%	55-150%
Factor XII activity level	28%	55-180%
Fibrinogen	586 mg/dL	200-400 mg/dL
Partial thromboplastin time (PTT) 1:1 immediate mix	90.2 seconds	25.6-35.9 seconds
Prothrombin time (PT) 1:1 immediate mix	15.0 seconds	12.0-14.2 seconds
Lupus anticoagulant activated partial thromboplastin time (aPTT)	139.7 seconds	29.8-44.4 seconds
Lupus anticoagulant activated partial thromboplastin time (aPTT) mixing study	118.2 seconds	29.8-44.4 seconds
Hexagonal phase assay	Positive	N/A
Von Willebrand factor antigen	465%	55-200%
Factor VIII inhibitor titer	1084 Bethesda Units (BU)	< 0.5 Bethesda Units (BU)

**Figure 1:**
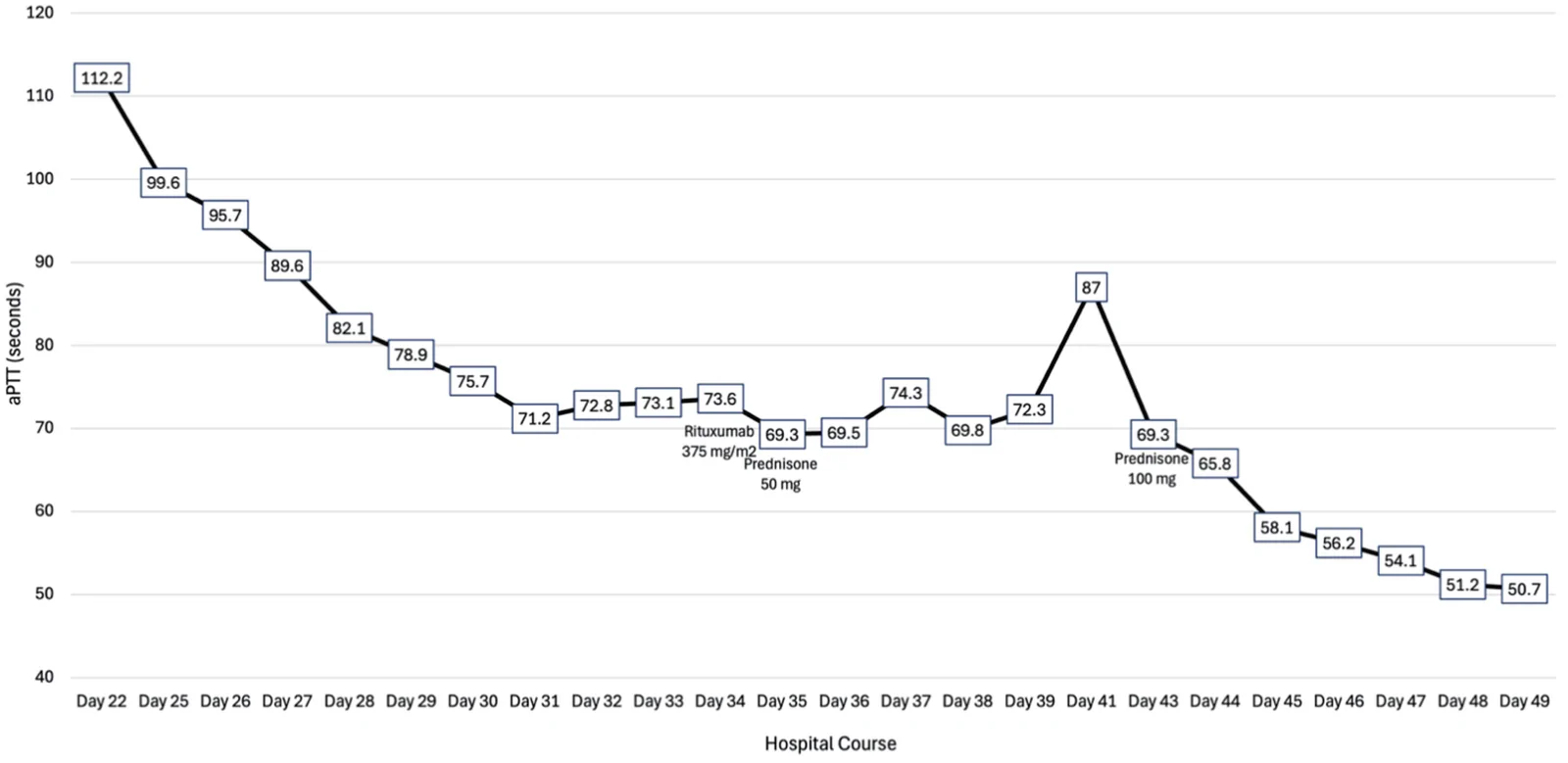
Graph of the patient’s aPTT trend throughout his hospital course. Day 25: prednisone 90 mg; Day 34: rituxan 375 mg/m2; Day 35: prednisone 50 mg; Day 43: prednisone 100 mg; Day 49: discharged

He was started on prednisone 1 mg/kg, and initially, his aPTT trended down until it plateaued 10 days into his steroid monotherapy. Figure 1 demonstrates his aPTT trend during his hospital course. Rituximab was initiated at 375 mg/m^2^ weekly for four weeks, and the prednisone dose was concomitantly lowered from 90 mg to 50 mg. Around 10 days later, on Day 41, the prednisone dose was increased to 100 mg due to an increase in aPTT. After receiving his third dose of rituximab, his aPTT reached 50.7 seconds, and the repeat Bethesda assay was 434 BU. While his iliopsoas hematoma size remained about the same throughout the hospitalization (8.3 x 6 cm), a repeat CTA scan during hospitalization showed markedly decreased attenuation, consistent with a resolving hematoma. He was safely discharged and continued prednisone treatment in the outpatient setting.

## Discussion

Our case presents a diagnostic challenge, since AHA and LAC prolong aPTT, and both produce an abnormal mixing study. The undetectable F VIII level should not be found with the LAC; thus, the diagnosis of acquired hemophilia A is achieved. Meanwhile, the abnormal dilute Russell’s Viper Venom Time (dRVVT) together with a confirmatory excess phospholipid test (hexagonal phase assay) confirms the finding of the lupus anticoagulant. The patient presented to the hospital with a prolonged aPTT of 103.9 seconds and a retroperitoneal hematoma. Throughout his hospitalization, the hematoma improved over time, but he remained easily bruised. His aPTT gradually decreased over time in response to dual steroid and rituximab therapy. The patient had no prior history of major bleeding or any autoimmune diseases. Upon review of his home medications, it was deemed that the antibodies that formed on F VIII were unlikely to be medication-induced.

The pathophysiology of AHA is thought to be mediated by polyclonal IgG antibodies with proteolytic properties, leading to the hydrolysis of F VIII into smaller fragments and therefore neutralizing them. This disrupts the intrinsic coagulation pathway, leading to spontaneous bleeding. In normal healthy individuals, low-titer anti-F VIII antibodies do exist, indicating that our immune tolerance, mediated by regulatory T cells, normally prevents the development of a pathologic immune response [6].

The concomitant presence of AHA and LAC found in our case highlights the importance of avoiding confounding factors during the workup of acquired hemophilia A [7, 8]. The acquired antibodies against F VIII in AHA predispose to hemorrhages, and conversely, the presence of lupus anticoagulant increases the risk of thrombosis. Therefore, patients having both autoantibodies can exhibit an array of symptoms ranging from thrombosis to hemorrhage, or both. It is crucial to distinguish between these two conditions, as both can prolong the aPTT, and neither would correct on mixing studies. In AHA, there would be markedly reduced F VIII activity, and immunoassays for anti-F VIII IgG4 would be positive, whereas LAC would show normal F VIII levels and no presence of anti-F VIII IgG4 [4]. Specialized coagulation testing, such as chromogenic Bethesda assays, hexagonal phase assay, and enzyme-linked immunosorbent assays, can be used to further identify both inhibitors and establish an accurate diagnosis [8]. In our patient, he had severely low F VIII activity, prolonged aPTT mixing time, highly increased Bethesda units, and a positive hexagonal phase assay, which confirmed the diagnosis of concurrent AHA and LAC.

The treatment of acquired hemophilia A can be divided into three categories: increasing F VIII levels, F VIII bypassing agents, and suppression of the inhibitor. The choice of treatment relies mainly on the presence or absence of active bleeding and the inhibitor titer levels. In patients with life-threatening bleeding and high inhibitor titer levels, bypassing agents like recombinant activated factor VII, activated prothrombin complex concentrate, or recombinant porcine F VIII can be used. Suppression of inhibitor levels can be achieved by immunosuppressive therapy, with the main goal of attaining remission. The limited existing literature regarding patients with coexisting AHA and LAC describes a combination therapy consisting of prednisone with cyclophosphamide or rituximab, which was found to be helpful for patients [7-8]. A prior 2012 study reviewed 331 patients who had AHA to check their response to immunosuppressive therapy. A total of 166 patients received steroids alone, 83 received steroids plus cyclophosphamide, 51 received rituximab-based regimens, and 31 received other regimens. It showed that steroids combined with cyclophosphamide resulted in more stable complete remission (70%), defined as undetectable inhibitor level, F VIII more than 70 IU/dL, and immunosuppression stopped, than steroids alone (48%) or rituximab-based regimens (59%) of patients [9-10].

An updated international recommendation from 2020 for AHA management outlined that those with F VIII <1% or >20 BU/ml can be started on steroids with rituximab or cyclophosphamide [9, 11]. This can be considered as first-line therapy for those who have high inhibitor titers [12]. Both AHA and LAC are caused by pathogenic autoantibodies that are produced by CD20+ B cells, and rituximab directly lowers CD20+ B cells, targeting the root cause of both conditions and making rituximab a favorable treatment [13-15]. Based on the guidelines, our patient was started on steroids and rituximab and showed a great response, as reflected in his down-trending aPTT and repeat inhibitor titers. Current recommendations for post-hospitalization include a 4-6-month glucocorticoid taper, which the patient completed [14].

## Conclusions

The presence of intercurrent AHA and LAC is extremely rare. This case highlights the diagnostic challenges with concomitant LAC in patients with new-onset bleeding and isolated prolonged aPTT. Any patient who presents with new-onset bleeding and isolated aPTT warrants further hematologic evaluation. In AHA, immunosuppressive therapy, including corticosteroids and rituximab, has been successfully used to achieve remission.
